# The Phosphorylation and Distribution of Cortactin Downstream of Integrin α9β1 Affects Cancer Cell Behaviour

**DOI:** 10.1038/srep28529

**Published:** 2016-06-24

**Authors:** Anette M. Høye, John R. Couchman, Ulla M. Wewer, Atsuko Yoneda

**Affiliations:** 1 Department of Biomedical Sciences, Faculty of Health and Medical Sciences, and Biotech Research and Innovation Centre (BRIC), University of Copenhagen, Copenhagen Biocenter, Ole Maaløes Vej 5, 2200 Copenhagen N, Denmark; 2 Laboratory of Genome and Biosignals, Tokyo University of Pharmacy and Life Sciences, 1432-1 Horinouchi, Hachioji-city, Tokyo 192-0392, Japan

## Abstract

Integrins, a family of heterodimeric adhesion receptors are implicated in cell migration, development and cancer progression. They can adopt conformations that reflect their activation states and thereby impact adhesion strength and migration. Integrins in an intermediate activation state may be optimal for migration and we have shown previously that fully activated integrin α9β1 corresponds with less migratory behaviour in melanoma cells. Here, we aimed to identify components associated with the activation status of α9β1. Using cancer cell lines with naturally occuring high levels of this integrin, activation by α9β1-specific ligands led to upregulation of fibronectin matrix assembly and tyrosine phosphorylation of cortactin on tyrosine 470 (Y470). Specifically, cortactin phosphorylated on Y470, but not Y421, redistributed together with α9β1 to focal adhesions where active β1 integrin also localises, upon integrin activation. This was commensurate with reduced migration. The localisation and phosphorylation of cortactin Y470 was regulated by Yes kinase and PTEN phosphatase. Cortactin levels influenced fibronectin matrix assembly and active β1 integrin on the cell surface, being inversely correlated with migratory behaviour. This study underlines the complex interplay between cortactin and α9β1 integrin that regulates cell-extracellular matrix interactions.

Integrins are cell surface, heterodimeric, transmembrane receptors mediating bidirectional signalling in both cell-cell and cell-extracellular matrix interactions^1^. In addition to being crucial for normal homeostasis, integrin cell surface expression and activation are important initiators and modulators of cancer cell behaviour^1^,^2^,^3^,^4^. Integrins are a pivotal part of the motility machinery for cells. β1 integrins can convert from a bent inactive, to an extended, active form in focal adhesions (FAs)^5^, suggesting the importance of conformational specificity and regulation in cell attachment and movement. Several members, but not all, of the integrin family have been extensively studied both at the conformational and the signalling level. Those are integrins such as αIIbβ3, αLβ2, and αXβ2, that are present on the surface of platelets or leukocytes where activation is important for platelet aggregation during hemostasis and thrombosis, or leukocyte migration and regulated immune response^6^,^7^. Moreover, the activation status of integrins may dictate recycling from the cell membrane^2^ further complicating the picture of integrin distribution and regulation.

Integrin α9β1 is important for postnatal survival, highlighted by the α9 knockout mouse^8^,^9^. Integrin α9β1 has been shown to play a role in the tumorigenesis and metastasis of several cancer types^9^. However, downstream signalling events from fully activated α9β1 integrins are largely unknown. We have previously reported that α9β1 likely exists in an intermediate activation state that can become fully activated upon treatment with Mn^2+^, a general integrin activator, or a β1-integrin activating antibody in G361 human malignant melanoma cells. The switch from intermediate to full activation resulted in altered adhesion and migration characteristics of the cells from a GTP-Rac- to Rho-associated protein kinase dependent manner, respectively^10^. The activation state of integrins is therefore important for melanoma cell behaviour. However, a paucity of data, particularly concerning α9β1 integrin, combined with highly complex regulatory and signalling networks provide an imperative to investigate the downstream signalling events and modulators of integrin activation.

Integrins lack intrinsic enzymatic activity and are therefore dependent on interactions with adaptor proteins, kinases and phosphatases for signalling. Activation of integrins can induce tyrosine phosphorylation of downstream multidomain adaptor proteins involved in regulating the cytoskeleton, such as cortactin^11^,^12^,^13^. The multidomain protein cortactin was first discovered as a major substrate of Src kinase^14^ and is important in actin cytoskeletal dynamics^15^.

Here, we find that α9β1 integrin full activation specifically leads to cortactin phosphorylation on Y470 in a Yes kinase- and PTEN phosphatase-dependent manner. Knockdown of cortactin results in loss of Mn^2+^ effects on integrin mediated functions such as migration and fibronectin (FN) matrix assembly, through altered integrin activation state. Importantly, cortactin phosphorylated on Y470, but not Y421, localises to FAs together with α9β1 upon integrin activation. Our data suggest that cortactin, and in particular phosphorylation of Y470, is important for cell behaviour where α9β1 is abundant.

## Results

### Full Activation of Integrins Leads to Increased Fibronectin Matrix Assembly in Cancer Cells

Integrins activated by Mn^2+^ promote a more rapid assembly of FN fibrils^16^ and melanoma cells have previously been reported to establish FN matrices^17^. The α9β1 integrin has been shown to be required for FN matrix assembly in lymphatic valve morphogenesis^18^. It is also an abundant integrin present on the cell surface of G361 human melanoma cells and on the human rhabdomyosarcoma cell line, RD^10^,^19^,^20^. Therefore, it was examined if full activation of integrins lead to altered FN matrix assembly in these cancer cells. Although integrin α5β1, regarded as the main integrin for FN matrix assembly in fibroblasts^21^, is expressed on the cell surface of G361 and RD cells^10^,^20^ we utilised α9β1-specific ligands in our studies to avoid α5β1-induced signalling.

**Figure 1:**
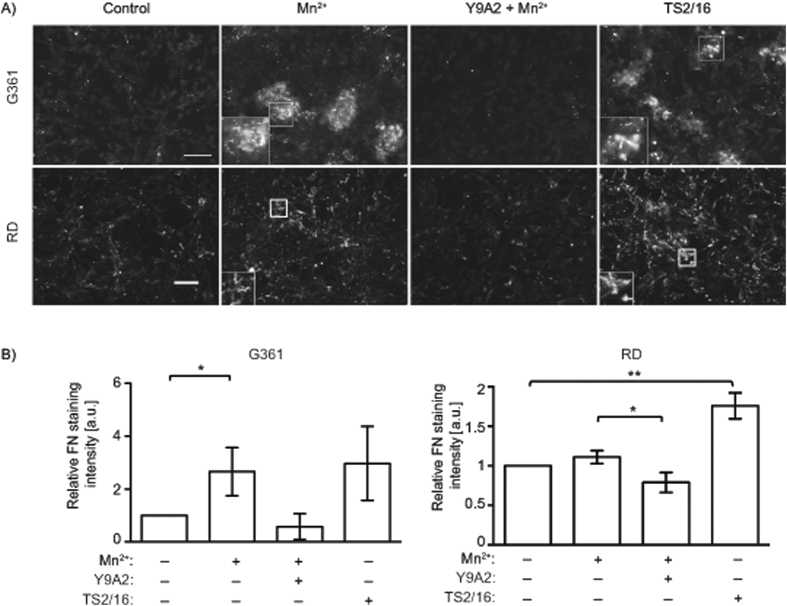
Integrin activation increases fibronectin matrix assembly. (**A**) Micrographs of G361 cells adherent to A12-Dis (upper panel) or RD cells adherent to TNfn3RAA (lower panel), in the absence or presence of Mn^2+^ (0.5 mM), or the α9β1-blocking antibody Y9A2 (10 μg/ml) in the presence of Mn^2+^ or treated with the β1-activating antibody TS2/16 (20 μg/ml) as indicated, and stained for FN. Scale bar: 75 μm for G361 cells, 50 μm for RD cells. Inserts are high-power micrographs (2x magnification) of boxed areas. (**B**) Quantitation of FN staining of micrographs in (**A**), fluorescence intensity was measured as described in Methods. Data from untreated control cells were set at 1.0. a.u. = arbitrary units. G361 data were analysed using unpaired two-tailed t-test and RD data were analysed using Mann-Whitney Rank Sum test.

G361 cells were seeded on the α9β1-ligand recombinant disintegrin domain of ADAM12 (A12-Dis), and treated with activators or inhibitors of integrin function (Fig. 1A). Activation of integrins by Mn^2+^ significantly increased FN matrix assembly compared to control cells (Fig. 1B). Moreover, the β1-activating antibody, TS2/16, also increased FN assembly while the α9β1 specific inhibitory antibody, Y9A2, blocked FN matrix assembly in response to Mn^2+^ (Fig. 1A,B). These results were confirmed using RD cells on the α9β1 integrin specific ligand the 3^rd^ fibronectin type III repeat of tenascin-C with mutation of the RGD sequence to RAA (TNfn3RAA)^22^,^23^ (Fig. 1A,B). Overall these results suggest that the activation status of integrin α9β1 affects FN matrix assembly in G361 and RD cells.

FN exists in two major forms, a cellular (cFN) and a plasma (pFN) form^24^,^25^. The α9β1 integrin binds to the EDA segment of FN present in cFN^26^. By PCR we could show that G361 cells express EDA-containing FN (Supplementary Fig. S1A). Interestingly, α9β1 binding to EDA-containing FN was recently shown to induce epithelial to mesenchymal transition in non-small cell lung carcinoma cells^27^. To evaluate if G361 cells assembled cFN in the absence of pFN, the experiment was performed with pFN-depleted serum. Indeed, G361 cells could assemble FN under this condition (Supplementary Fig. S1B), suggesting that the cells assemble cFN matrix, the isoform to which α9β1 integrin binds. Western blotting of the deoxycholate (DOC) insoluble FN of G361 cells further confirmed that Mn^2+^ treatment increases the amount of FN EDA (cFN) compared to untreated G361 cells (Supplementary Fig. S1C,D). These results indicate that the full activation state of α9β1 is more potent than its intermediate state (indicated by adhesion to cognate ligands) to assemble FN matrix in G361 cells.

### Tyrosine Phosphorylation of Cortactin Accompanies Full Activation of α9β1 Integrin

**Figure 2:**
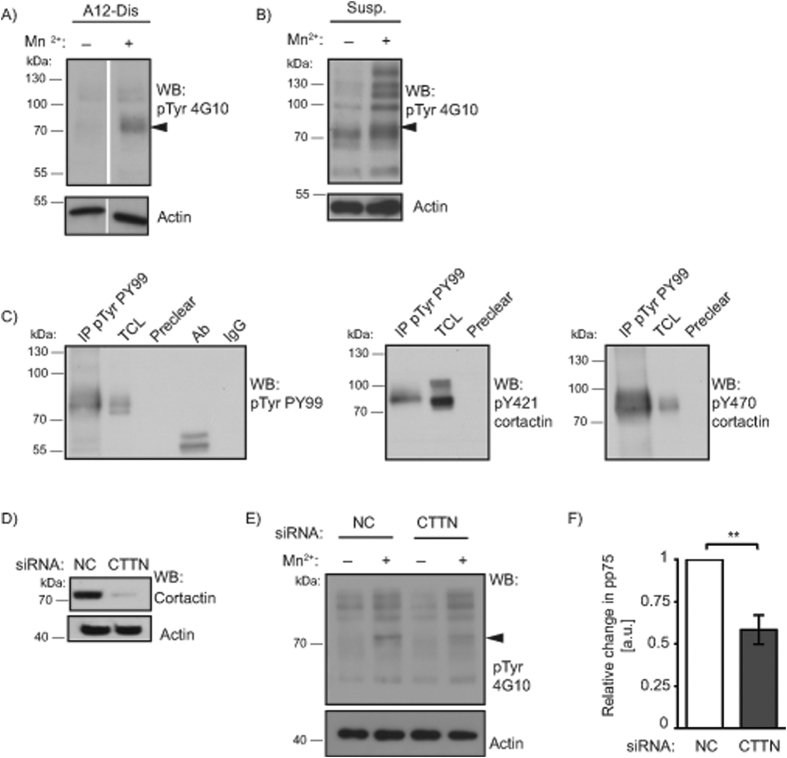
Integrin activation results in tyrosine phosphorylation of a 75 kDa protein, identified as cortactin. (**A**) G361 total cell lysates (TCLs) after adhesion to A12-Dis in the absence or presence of Mn^2+^ (1 mM) were analysed by WB using an anti-phosphotyrosine (pTyr) antibody (4G10). Full activation of integrins by Mn^2+^ leads to tyrosine phosphorylation of a 75-kDa (pp75) protein (arrowhead) compared to untreated cells. Total actin was used as loading control. (**B**) G361 cells in suspension with or without Mn^2+^ (3 mM) blotted for pTyr (4G10) and actin. (**C**) Immunoprecipitation (IP) with anti-pTyr antibody (PY99) of TCL from G361 cells in suspension treated with Mn^2+^ (3 mM). Samples were analysed by WB. TCL, preclear beads, antibody (Ab) alone, and mouse IgG (IgG), were included as controls. WBs were probed for pTyr (PY99), pY421 cortactin, and pY470 cortactin as indicated. (**D**) WB analysis of G361 TCL after 48 hours transfection with negative control (NC) or cortactin (CTTN) siRNA using cortactin antibody. (**E**) TCL from NC or CTTN siRNA treated cells was probed against pTyr (4G10) and actin. (**F**) Quantitation of (**E**) for relative levels of p75 tyrosine phosphorylation after Mn^2+^ treatment. Data from Mn^2+^ treated NC cells were set at 1.0. Data were analysed using unpaired two-tailed t-test.

In addition to having an effect on FN matrices, integrin activation on α9β1-binding substrates leads to decreased migration and increased cell spreading^10^,^19^, but little is known about components involved in signalling from the active integrin. Therefore, changes in protein tyrosine phosphorylation in G361 cells were analysed after adhesion to A12-Dis in the absence or presence of Mn^2+^. Increased tyrosine phosphorylation of a 75-kDa protein (pp75) was seen in lysates of Mn^2+^ stimulated cells compared to unstimulated controls (Fig. 2A). pp75 was also observed in cell lysates from cells in suspension treated with Mn^2+^ compared to untreated (Fig. 2B), suggesting the event is independent of ligand ligation, requiring only the active conformational state of the integrins. To identify pp75, tyrosine phosphorylated proteins were immunoprecipitated (IP) with the anti-phosphotyrosine antibody (PY99) from G361 cells in suspension treated with Mn^2+^ and eluted with phenyl phosphate. The eluted sample was subjected to western blotting for candidate proteins (Supplementary Fig. S2). Phosphorylated cortactin Y421 (pY421) and Y470 (pY470) was positively identified by IP (Fig. 2C), consistent with a 75 kDa mass. siRNA knockdown experiments confirmed that pp75 was cortactin (Fig. 2C–F). Knockdown of cortactin led to a statistically significant reduction of pp75 in response to integrin activation (Fig. 2F).

### Cortactin Y421 and Y470 Residues Are Differentially Phosphorylated Upon Full Activation of Integrins

**Figure 3:**
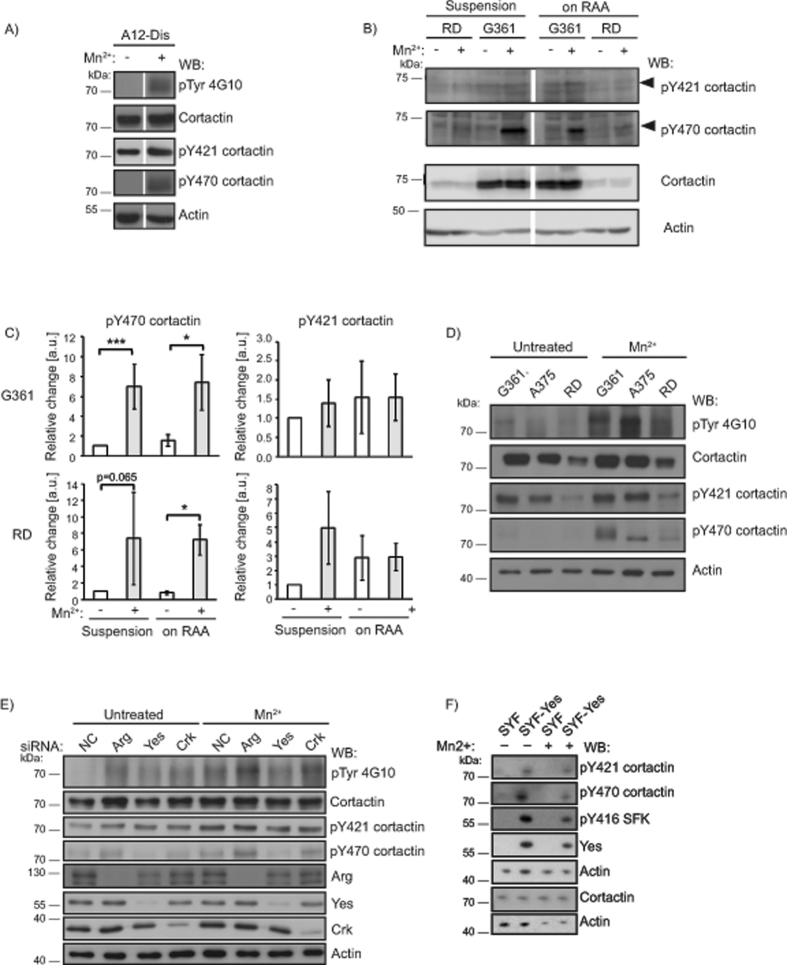
Cortactin Y421 and Y470 are differentially phosphorylated upon full activation of integrins. (**A**) WB of G361 TCL from cells in suspension or after adhesion to A12-Dis, in the absence or presence of Mn^2+^ (1 mM). (**B**) WB of RD and G361 TCL from cells in suspension or after adhesion to TNfn3RAA, in the absence or presence of Mn^2+^ (1 mM). (**C)** Quantitation of (**B**) for relative levels of cortactin Y470 and Y421 tyrosine phosphorylation after Mn^2+^ treatment. Data from untreated NC cells were set at 1.0. Data were analysed using Mann-Whitney Rank Sum test. (**D**) WB of TCL from G361, A375 and RD cells after adhesion to A12-Dis in the absence or presence of Mn^2+^ (1 mM). (**E**) G361 cells were transfected with NC, Arg, Yes, or Crk siRNA, and changes in cortactin tyrosine phosphorylation of Y421 and Y470 upon knockdown were evaluated by WB. (**F**) WB of TCL from SYF cells and SYF cells re-expressing Yes (SYF-Yes) that were either left untreated or subjected to Mn^2+^ treatment. This WB also shows that the antibodies recognising phosphorylated cortactin Y421 and Y470 discriminate phosphorylated from non-phosphorylated cortactin. Blots were analysed and probed for pTyr (4G10), cortactin, pY421 cortactin, pY470 cortactin, pY416 SFK, v-Src, Arg, Yes, Crk, and actin, as indicated.

Cortactin has multiple tyrosine residues that can undergo phosphorylation. Murine cortactin residues Y421/Y466/Y482, corresponding to human Y421/Y470/Y486^28^,^29^, have been regarded as the key tyrosine residues of cortactin undergoing phosphorylation. For clarity, we use the human cortactin residue numbering. Cortactin Y421 and Y470, but not Y486, phosphorylation have been shown to be crucial for actin polymerisation in invadopodia and tumour cell invasion^30^. Therefore, we evaluated the tyrosine phosphorylation patterns of cortactin Y421 and Y470. Unlike the constant levels of pY421, pY470 was upregulated upon activation of integrins by Mn^2+^ in cells in suspension or adherent to A12-Dis or TNfn3RAA (Fig. 3A–C). This suggests that a pool of cortactin Y421 is constitutively phosphorylated in these cells while phosphorylation of cortactin Y470 is sensitive to integrin activation states.

The human melanoma cell line A375 also expresses α9β1 on the cell surface^10^,^20^. G361, A375, and RD were seeded on A12-Dis coated substrates in the absence or presence of Mn^2+^. pp75 and pY470 cortactin were increased after integrin activation in all three cell lines (Fig. 3D), suggesting that phosphorylation of Y470 upon full integrin activation occurs in multiple cell lines expressing α9β1 integrin.

### Integrin Activation Signals Yes-Mediated Cortactin Y470 Phosphorylation

Cortactin was first identified as a Src substrate^14^,^31^. To identify the kinase responsible for phosphorylation of Y470, the presence of kinases known to phosphorylate cortactin was evaluated by western blotting of G361 total cell lysates. FAK, Pyk2, Yes, Crk, Arg, and Src were detected, but not Fyn and Hck (Supplementary Fig. S3A). Src was only detectable in G361 cells lysates after long exposures, therefore we focussed our attention on the other kinases present. Active FAK can directly bind and phosphorylate cortactin^11^,^32^. Therefore the role of FAK was evaluated. However, FAK siRNA did not reduce pY470 upon full activation of integrins by Mn^2+^ nor did it have an effect on pY421 (Supplementary Fig. S3B). These results suggest FAK is not involved in phosphorylating Y470. Next, knockdown of the Arg, Yes, and Crk kinases were evaluated. Only Yes knockdown led to a decrease in pp75 and pY470 levels (Fig. 3E). Intriguingly, Arg knockdown showed increased pY470, suggesting a complex regulation of cortactin phosphorylation.

Of the nine Src family kinase members (SFK) Src, Yes, and Fyn are ubiquitously expressed. Therefore, the Src/Yes/Fyn (SYF)^33^ triple knockout mouse embryonic fibroblasts (MEFs) and SYF fibroblasts re-expressing Yes (SYF-Yes) were investigated to determine if Yes kinase could be responsible for pY470. The SYF cells exhibited undetectable basal levels of pY470, as well as, minimal response upon full integrin activation (Fig. 3F). The reintroduction of Yes (SYF-Yes cells) on its own or in combination with Mn^2+^ facilitated cortactin phosphorylation of both Y421 and Y470 (Fig. 3F). This is consistent with cortactin being a prominent Src family substrate, where Src and Yes are the most closely related SFKs^34^. Taken together, these results indicate that α9β1 integrin activation signals Yes-mediated phosphorylation of cortactin Y470 in G361 cells.

### Cortactin Knockdown Increases Fibronectin Matrix Assembly

**Figure 4:**
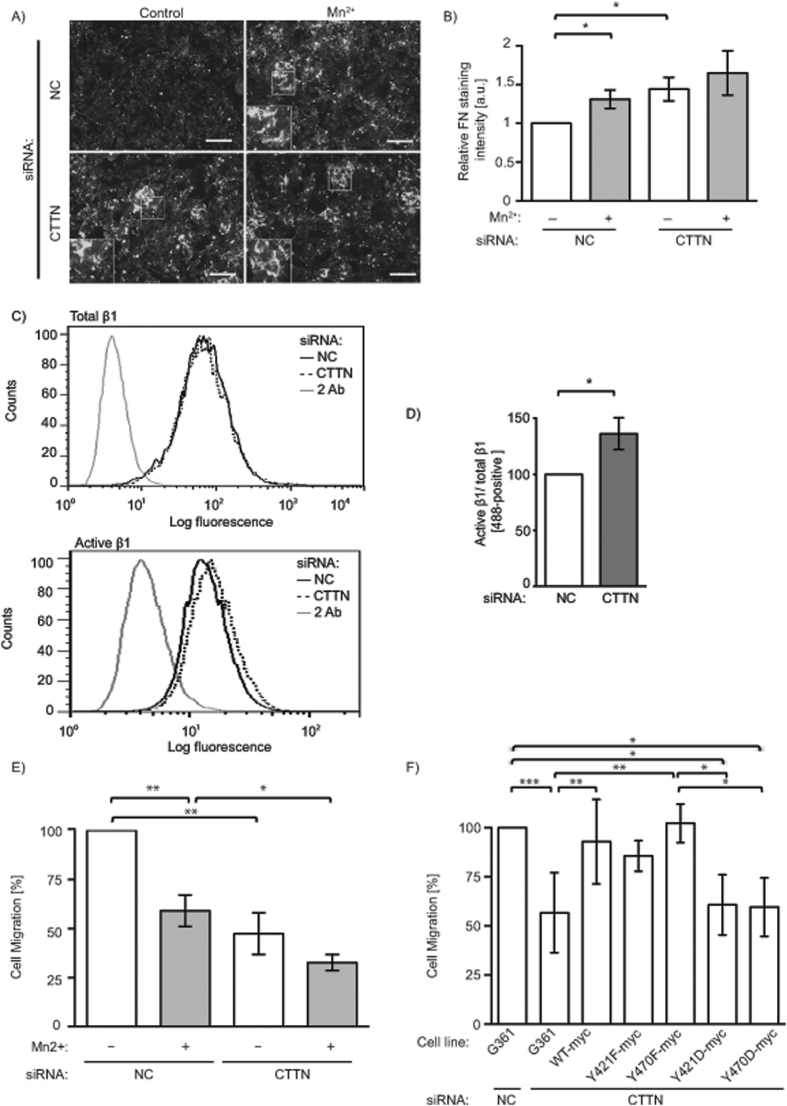
Knockdown of cortactin increases FN matrix and active β1 integrin levels, while decreasing migration on A12-Dis. (**A**) Micrographs of G361 cells attached to A12-Dis treated with NC or CTTN siRNA in the absence or presence of Mn^2+^ (0.5 mM), and stained for FN. Inserts are high-power micrographs (2x magnification) of boxed areas. Scale bars = 75 μm. All micrographs are representative. (**B**) Quantitation of (**A**). Data were analysed using unpaired two-tailed t-test. (**C**) Representative FACS profiles of total β1 (MAB1959, top graph) and active β1 (12G10) integrin (bottom graph) on the surface of G361 cells. (**D**) Quantitation of (**C**). Data were analysed using unpaired two-tailed t-test. (**E**) Cell migration of cortactin siRNA-treated G361 cells in the absence or presence of Mn^2+^ (1 mM). Data were analysed using unpaired two-tailed t-test. (**F**) Parental G361 were treated with NC or CTTN siRNA. G361 cells stably transfected with WT-myc, Y421F-myc, Y470F-myc, Y421D-myc, or Y470D-myc where indicated, were double transfected with CTTN siRNA and their respective plasmid 48 hours before migration. (**E,F**) Data are an average of three independent experiments performed in duplicate. Data were analysed using one-way ANOVA with Tukey’s multiple comparison test. (**B–F**) Data from untreated NC cells were set to 1.0 or 100.

Integrin interactions with the cytoskeleton are necessary for FN matrix assembly at the cell surface^35^. Besides being involved in actin cytoskeleton dynamics, cortactin has been implicated in secretion and deposition of FN^36^. Cortactin siRNA was next employed to evaluate if cortactin influenced FN matrix assembly in G361 cells seeded on A12-Dis. As expected, treatment with Mn^2+^ led to a significant increase in FN matrix in negative control siRNA (NC) treated cells compared to untreated NC cells (Fig. 4A,B). Importantly, even without Mn^2+^ treatment cortactin knockdown cells also significantly increased FN matrix assembly compared to untreated NC cells (Fig. 4A,B). Activation of integrins by Mn^2+^ gave no additional increase of FN matrix assembly upon cortactin knockdown.

As a control for cortactin contribution to FN assembly, we analysed cortactin knockout (KO) MEFs seeded on A12-Dis. Both wild-type (WT) and KO cells formed a FN matrix (Supplementary Fig. S4A), with a tendency towards more FN staining in KO cells compared to WT, but this was not statistically significant (Supplementary Fig. S4B). This may be due to lower amounts of cortactin in MEFs compared to G361 cells and/or the absence of α9β1 integrin^37^. These results suggest that cortactin is a negative regulator of FN matrix assembly in G361 cells, while having less effect on MEFs, irrespective of integrin activation by Mn^2+^.

### Cortactin Knockdown Increases Active β1 Integrin on the Cell Surface

Since cortactin knockdown regulates integrin-mediated events leading to increased FN assembly we investigated the possibility that cortactin could have an effect on the activation levels of β1 integrins. The level of total cell surface β1 remained unchanged upon cortactin knockdown (Fig. 4C). However, knockdown of cortactin showed a significant increase in the cell surface levels of active β1 detected by the 12G10 antibody in FACS analysis (Fig. 4D). This suggests that cortactin negatively regulates active β1 integrin levels on the surface of G361 cells.

### Consistent with β1 activation, Migration of G361 Cells is Decreased Upon Cortactin Knockdown

Previously it has been shown that the presence of Mn^2+^ or an α9β1 blocking antibody led to decreased G361 migration on α9β1 ligands^10^,^19^. Therefore, we investigated if cortactin protein levels could affect G361 cell migration. NC or cortactin knockdown G361 cells were allowed to migrate on A12-Dis in the presence or absence of Mn^2+^. Full activation of integrins by Mn^2+^ in NC cells lead to a significantly less migratory behaviour compared to untreated NC cells. Knockdown of cortactin also significantly reduced G361 cell migration compared to NC and it was comparable level with that of Mn^2+^ treated NC cells (Fig. 4E). Moreover, the combination of cortactin knockdown and integrin activation by Mn^2+^ further reduced migration below that of Mn^2+^ alone for NC cells. There was no significant difference between the migration of cortactin knockdown cells without or with Mn^2+^ treatment, suggesting that cortactin is essential for the reduction in migratory behaviour seen upon integrin activation (Fig. 4E).

### Phosphorylation of  Y470 in Cortactin is Key to Reduced Migration in Melanoma

To evaluate the contribution of cortactin Y421 and Y470 phosphorylation in migration on A12-Dis, stable cell lines expressing murine myc-tagged wild-type (WT-myc), phosphorylation-resistant cortactin Y421F (Y421F-myc) and cortactin Y470F (Y470F-myc), or phosphomimetic cortactin Y421D (Y421D-myc) and cortactin Y470D (Y470D-myc) mutants, were prepared. To avoid effects caused by endogenous cortactin, stably expressing cells were transiently transfected with cortactin siRNA targeting human cortactin only, 48 h before migration assays (Supplementary Fig. S5). Overexpression of WT-myc cortactin rescued the migration to the same level as untreated NC. Cells overexpressing phosphorylation-resistant cortactin Y470F-myc were also significantly more migratory compared to cortactin knockdown G361 cells, while the migration of cells expressing cortactin Y421F-myc was not significantly different to cortactin knockdown alone (Fig. 4F). However, the phosphomimetic cortactin Y421D-myc and Y470D-myc reduced migration significantly compared to NC and compared to cortactin Y470F-myc (Fig. 4F), suggesting that the phosphorylation status of cortactin Y470 was more important than Y421 for migration of G361 cells on A12-Dis. Therefore, cortactin regulates migration in G361 melanoma cells which in turn is dependent on its phosphorylation status.

### Phosphorylated Cortactin Y470 Redistributes Together With α9β1 to Focal Adhesions Upon Integrin Activation

**Figure 5:**
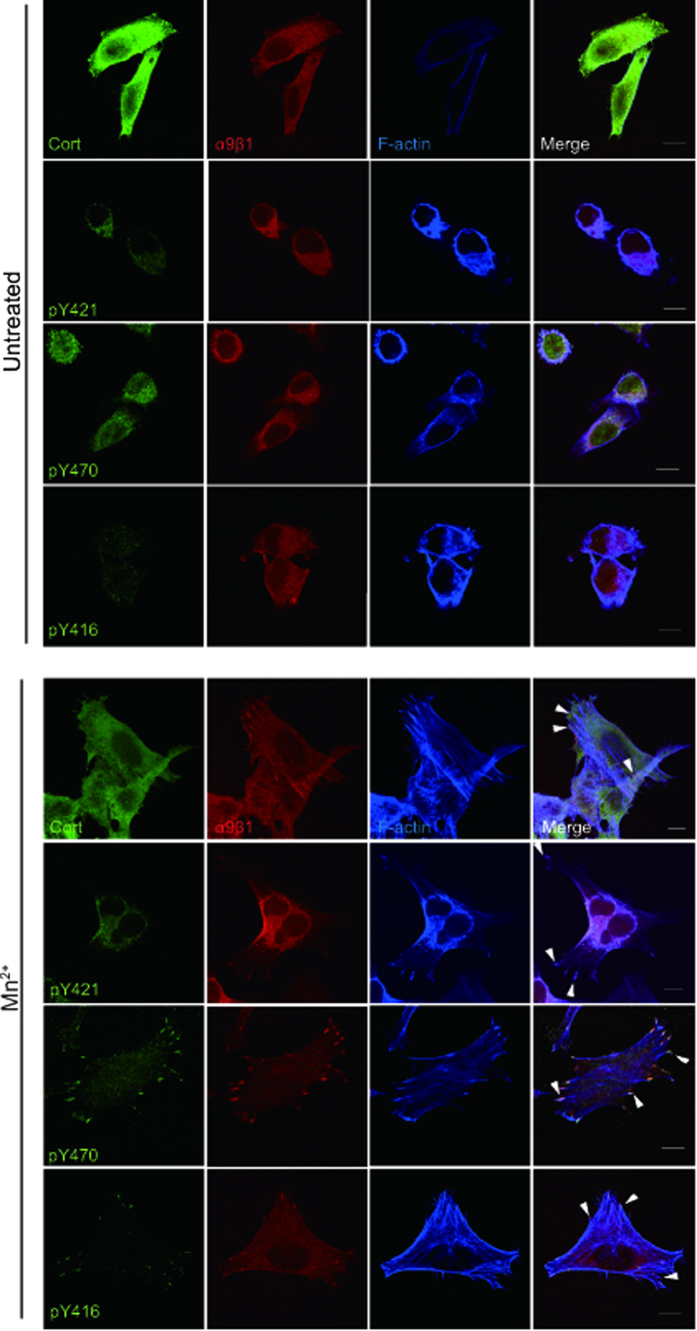
Integrin α9β1, pY416 SFK and cortactin pY470, but not pY421, localises to focal adhesions upon integrin activation. Representative confocal micrographs of G361 cells seeded on A12-Dis. Cells were stained for cortactin (cort), pY421 cortactin (pY421), pY470 cortactin (pY470), pY416 SFK (pY416), α9β1 (red), and F-actin (blue). Arrowheads indicate focal adhesion structures. Scale bars = 10 μm.

We have shown previously that α9β1 localises to FAs upon full activation of integrins^10^. To evaluate if cortactin localises to FAs, G361 cells were seeded on A12-Dis. Strikingly, pY470 cortactin and phosphorylated Y416 Src family kinases (pY416 SFK), but not pY421 cortactin, specifically redistributed to FAs (arrowheads) along with α9β1 upon full activation of integrins (Fig. 5). Total cortactin staining was uniform in these cells, so that any subpopulation in FAs was not discretely visible.

### The Phosphorylation Status of  Y470 in Cortactin is Affected by Integrin α9, but not by α4

To further investigate if tyrosine phosphorylation of cortactin was involved in α9 integrin signalling, α9 or its closely related subfamily member α4 integrin were knocked down by siRNA and validated by FACS (Supplementary Fig. S6A,B). Surprisingly, knockdown of α9 resulted in increased pY470 on A12-Dis substrates, both in the absence and presence of Mn^2+^ (Supplementary Fig. S6C). The pY421 levels were unaffected by α9 knockdown. Moreover, knockdown of α4 did not affect the levels of pY421 or pY470.

Since knockdown of α9 integrin increased levels of pY470, we hypothesised that one or more tyrosine phosphatases might affect tyrosine phosphorylation of Y470 in G361 cells. We took advantage of the broad-spectrum phosphatase inhibitor pervanadate (PV) to evaluate whether a phosphatase was involved in phosphorylation of cortactin in G361. Tyrosine phosphorylation of cortactin and its Y470 residue was seen after 30 min in suspension with 1 μM PV, while 10 μM PV gave a substantial increase of pY470, pY421, and of pY416 SFK (Supplementary Fig. S6D). Ten micromolar PV was needed to observe increased pY421. This suggests that a tyrosine phosphatase is important for the regulation of cortactin in G361 cells, and that cortactin Y470 phosphorylation is more sensitive than Y421 to tyrosine phosphatase inhibition.

### PTEN Affects Localisation and Phosphorylation of Cortactin Y470

**Figure 6:**
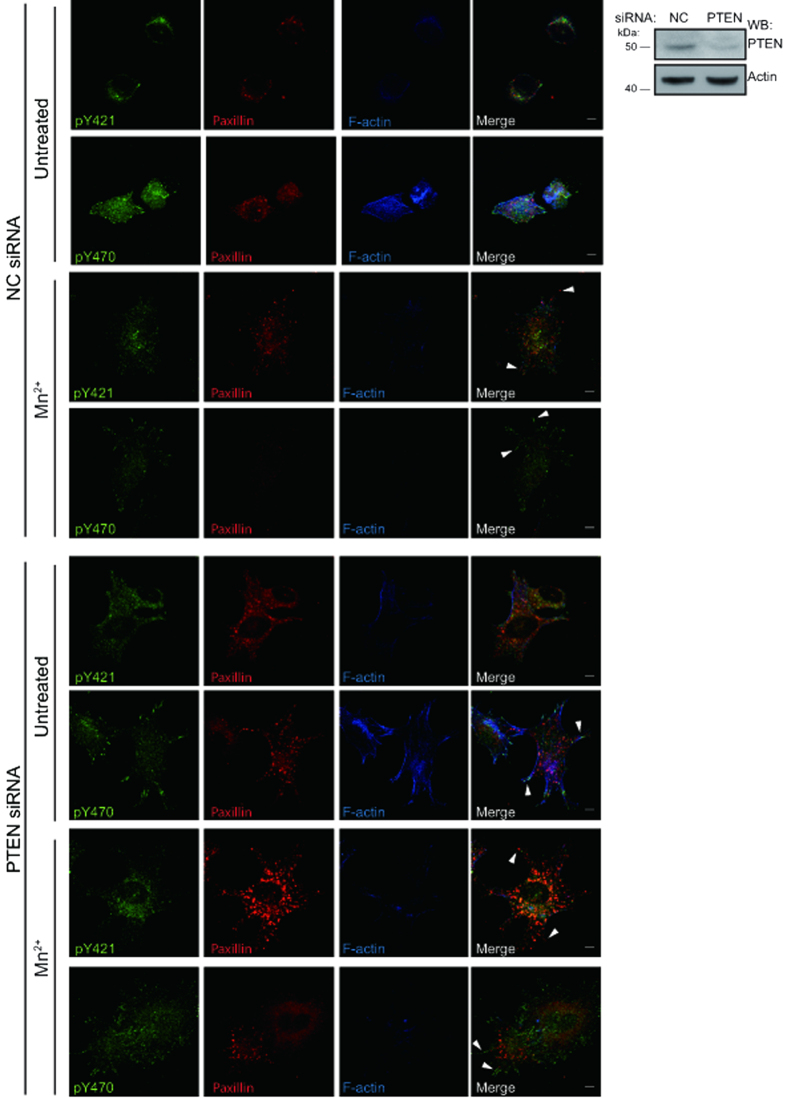
Knockdown of PTEN drives cortactin pY470 into focal adhesions regardless of integrin activation. Representative confocal micrographs of parental G361 with NC or PTEN siRNA seeded on TNfn3RAA. Cells were stained for pY421 cortactin (pY421), pY470 cortactin (pY470), paxillin (red), and F-actin (blue). Scale bars = 10 μm. WB analysis of G361 TCL after 48 hours transfection with NC or PTEN siRNA using PTEN antibody.

The tumour suppressor and phosphatase, phosphatidylinositol 3,4,5-trisphosphate 3-phosphatase and dual-specificity protein phosphatase (PTEN), has been shown to affect integrin-mediated cell spreading, FA formation and migration^38^,^39^. PTEN expression has also previously been linked to FN expression^40^. To investigate the potential role of PTEN in the α9β1 integrin-cortactin pathway we performed siRNA knockdown of PTEN and seeded the cells on TNfn3RAA (Fig. 6). Increased cell spreading and redistribution of cortactin pY470 to focal adhesions, but not cortactin pY421, was seen in PTEN siRNA treated G361 cells, thereby, phenocopying Mn^2+^ treated G361 cells (Fig. 6). Full integrin activation of PTEN knockdown cells on TNfn3RAA showed a similar phenotype to untreated PTEN knockdown cells. These results suggests that PTEN regulates cortactin pY470 downstream of integrin α9β1.

## Discussion

Cortactin is recognised for its ability to regulate the submembranous cytoskeleton, and has roles in invadopodia of some tumour cells^41^. The human G361 melanoma cell line is invasive, and as we showed previously has a high level of α9β1 integrin on the cell surface^10^. Recently, mRNA levels of integrin α9 were shown to be higher in primary human melanoma samples, compared to normal skin samples^42^. Integrin α9 mRNA is negatively regulated by miRNA-125b, which is downregulated in primary melanoma^42^. The human rhabdomyosarcoma cell line, RD, also expresses a very high level of cell surface α9 integrin^37^. In rhabdomyosarcoma, Notch-induced integrin α9 increases cell adhesion and invasion^43^. Here, for the first time, linkage of this integrin to cortactin is demonstrated (Supplementary Fig. S7). This impacts several functions of these cells. Under normal culture conditions, G361 cells are readily adherent to α9β1 ligands, but remain migratory, with little development of FAs and associated microfilament bundles. However, when challenged with Mn^2+^ to activate integrins, these cells form FAs on α9β1 ligands in which the integrin, phosphorylated cortactin and SFKs concentrate. Consistent with this, migration is reduced, and the inverse relationship between FA assembly and migration on planar substrates has been known for many years^44^.

Exogenous activation of the α9β1 integrin led to increased phosphorylation of cortactin on Y470, though levels of pY421 were largely unchanged (Fig. 3A–C). It has been suggested that Y421 is a priming site for the docking of the SH2 domain of Src, important for the subsequent phosphorylation of Y470^29^,^45^. The observed constant phosphorylation of cortactin Y421 in G361 cells, independent of adhesion and activation, does not contradict these data. However, it appears that the phosphorylation of Y470 may localise cortactin to FAs (Fig. 5), suggestive of interactions through the phosphotyrosine residue with other FA components. Very few reports have investigated the specific contributions of each cortactin tyrosine residue, but a previous study showed that cortactin Y470 was selectively phosphorylated downstream of overexpression of fibroblast growth factor receptor 1 in HEK293 cells^46^. Moreover, in epidermal growth factor induced phosphorylation of cortactin, Y470 was suggested to be the preferred tyrosine site for interactions with cofilin^30^. This indicates that differential phosphorylation of cortactin is another regulatory step important for selective binding of cortactin interaction partners and functional outcomes such as cell spreading and migration. Our data support that Y421 and Y470 can be differentially phosphorylated.

The importance of cortactin phosphorylation is underscored by the examination of mutants where the key tyrosine residues were mutated either to phenylalanine or aspartate. The Y-F mutants had the same impact on migration as WT cortactin when expressed in cells depleted of endogenous protein. In contrast, the phosphomimetic mutant proteins were incapable of restoring migration in the G361 cells (Fig. 4F). This is commensurate with the hypothesis that phosphorylation, particularly of Y470, is an important down-regulator of migratory activity. In the untreated, malignant and invasive cells, this phosphorylation is low, but is increased by exogenous integrin activation. The net effect is to maintain the cell in an optimal migratory state, where the α9β1 integrin supports adhesion sufficient for locomotion, but insufficient for stable adhesion characterised by FA assembly. Down-regulation of cortactin, or its phosphorylation alters this balance in favour of stronger adhesion, also achieved by exogenous integrin activation. These results are in agreement with a study showing that hyperphosphorylated cortactin suppressed migration in gastric cell lines^47^. Tyrosine phosphorylation of cortactin is generally associated with the recruitment of proteins binding to the SH3- or the proline-rich domain, leading to activation of the Arp2/3 complex, resulting in lamellipodial protrusion and migration^41^. However, loss of cortactin did not affect adhesion or migration on FN^48^, but was shown to decrease adhesion and migration on poly-lysine-D or uncoated surfaces, respectively^36^,^49^. The results from these studies indicate that cortactin mediated-adhesion and migration may be cell type- or ligand-specific.

Phosphorylation of cortactin was considerably enhanced by treatment of the cells with phosphatase inhibitor (Supplementary Fig. S6D). The evidence suggests that Yes kinase, a close relative of Src, is responsible for Y470 phosphorylation of cortactin (Fig. 3E–F). In addition, the phosphatase PTEN affects phosphorylation and distribution of cortactin Y470 to FAs (Fig. 6). Pervanadate treatment substantially increased cortactin phosphorylation but it appears that the Y421 and Y470 residues had different sensitivities to phosphatase inhibition (Supplementary Fig. S6D). Importantly, it suggests that in G361 cells, integrin α9β1 functions to maintain cortactin Y470 phosphorylation at low levels, which is optimal for migration.

Feedback from cortactin to integrins is also apparent from our current data. Reductions in cortactin in the tumour cells by siRNA led to a small, but reproducible and significant increase in the level of active integrin, as detected by conformation-specific β1 integrin antibodies (Fig. 4D). Control siRNA had no effect. It is likely that this effect is mediated through changes in integrin recycling, known to be important in cell migration and invasion^50^,^51^. This is related to the finding that cortactin depletion also resulted in reduced migration (Fig. 4E).

Matrix assembly is often minimal in transformed cells, and G361 melanoma cells are no exception. However, a FN matrix is supported by integrin, α9β1. Changes in the expression level or in the fibrillar organisation of FN have been shown to be important for cancer progression by affecting processes such as cell migration, growth, and tumour angiogenesis^21^. It has been shown previously that the EDA repeat of FN interacts with this integrin^52^, and we could show expression of EDA^+^ FN at the mRNA and protein levels by G361 cells (Supplementary Fig. S1). Reminiscent of the migration data, either cortactin depletion, or integrin activation by Mn^2+^ increased matrix assembly (Figs 1 and 4A,B). Matrix assembly is often a characteristic of less motile cells, so that the minimal levels seen in untreated G361 cells is unsurprising, and consistent with the migration data. In contrast to the G361 cells, cortactin KO fibroblasts seeded on A12-Dis show no significant alteration in FN matrix assembly, but do demonstrate that this cytoskeletal protein is not absolutely required (Supplementary Fig. S4). In WT MEFs, however, the α9β1 integrin is not expressed^37^, and/or cortactin level is lower than cancerous cells as the gene is often amplified in various human cancers^15^, so that matrix assembly is presumably governed by other A12-Dis binding-integrins such as αvβ3. Further experiments will be required to address whether cortactin is required for FN matrix assembly mediated by specific integrins and whether α9β1 integrin affects α5β1 integrin-mediated FN matrix assembly.

In summary, cells where integrin α9β1 is highly expressed may be geared towards a migratory phenotype. The integrin is sufficiently active to support adhesion compatible with migration, and in part this is mediated by control of cortactin function. By stimulating integrin activation, changes in cortactin status and distribution slow migration and enhance matrix assembly. Whether this represents an opportunity for intervention remains to be seen, but does exemplify how a malignant cell type such as melanoma regulates cytoskeleton-integrin-matrix interactions to optimise the invasive state.

## Materials and Methods

### Cell Culture

The human malignant melanoma cell lines G361 and A375, the human rhabdomyosarcoma cell line RD, Src/Yes/Fyn (SYF) −/− mouse embryonic fibroblasts (MEFs) were obtained from American Type Culture Collection. Cortactin WT and KO MEFs were a kind gift from Dr. Klemens Rottner (University of Bonn, Germany). G361 was cultured in McCoy’s 5A medium (Invitrogen) containing 10% foetal bovine serum (FBS) (Hyclone). SYF, cortactin WT and KO MEFs were cultured in Dulbecco’s modified medium (Invitrogen) containing 5% FBS. Cells were grown at 37 °C in a 5% CO_2_ humidified atmosphere. Stable G361 cell lines expressing cortactin constructs (WT-myc, Y421F-myc, Y470F-myc, Y421D-myc, or Y470D-myc) were established by selection with 500 μg/ml G418.

### Antibodies

The following mouse monoclonal antibodies were used: anti-phosphotyrosine PY99 (Santa Cruz Biotechnology), anti-phosphotyrosine (4G10), anti-α9β1 antibody (Y9A2), anti-integrin α4 (HP2/1), anti-total β1 (MAB1959), anti-active β1 (12G10), anti-myc tag (4A6), anti-v-Src (OP07), anti-PTEN (6H2.1), anti-pan-actin (all from Millipore), activating β1-integrin antibody (TS2/16, Thermo Scientific), anti-Yes, anti-Fyn, anti-Hck, anti-Crk (all from BD Biosciences), anti-cellular/EDA fibronectin antibody (FN-3E2, Sigma). The following rabbit polyclonal antibodies were used: anti-cortactin (ab11066 from Abcam, for G361 lysates) and anti-cortactin phospho-Y470 (ab51703, from Abcam, or sc-101661 from Santa Cruz), anti-cortactin phospho-Y421 and anti-Src family kinases (SFK) phospho-Y416 (both from Cell Signaling), anti-arg, anti-Pyk2, anti-FAK (all from Millipore), anti-FAK phospho Y397 (Invitrogen), and anti-fibronectin (R2/7, made against bovine plasma fibronectin^53^). Secondary antibodies used were fluorochrome-conjugated antibodies (Alexa Fluor 488 and 546, Invitrogen) and horseradish peroxidase (HRP)-conjugated antibodies (Dako).

### siRNA Oligonucleotides and Transfection Reagents

siGENOME SMARTpools against cortactin (M-010508-00), Arg/ABL2 (m-003101-02-00), Crk (m-010503-03-00), Yes (m-003184-03-00), PTK2/FAK (m-003164-02-00), α9 (m-008005-01-00) (all from Dharmacon), Silencer Select siRNA against α4 (4390824, Invitrogen/Ambion) and PTEN (PS130240361-011, Sigma), were transfected using Lipofectamine 2000 (Invitrogen) according to the manufacturer’s instructions. AllStars negative control (NC) siRNA (1027281) (Qiagen) was used as negative control. Cells were transfected for 48 h before performing experiments.

### Plasmids and Transfection Reagents

A myc-tagged murine cortactin wild-type (WT) cDNA in pCMV Tag5B was a kind gift from Dr. Steve Zhan (University of Maryland, USA). Mutations encoding Y421F, Y421D, Y470F, and Y470D, were introduced by PCR using a plasmid carrying a wild-type cDNA as a template and primer sets Y421F forward (5′–TTTGAGGATGCAGCTCCGTTCAAGGC–3′)/Y421F reverse (5′– GATGGGGCTGGAGGGTGGTCT–3′), Y421D forward (5′–GATGAGGATGCAGCTCCGTTCAAGGC–3′)/Y421F reverse, Y470F forward (5′–TTCGAGACTACAGAGGCTCCTGGC–3′)/Y470F reverse (5′–CACGGGCTCTGATGTATAGGTCAGG–3′), and Y470D forward (5′-GACGAGACTACAGAGGCTCCTGGC-3′)/Y470F reverse, respectively. PCR products were digested with *Dpn* I (Invitrogen), then phosphorylated by T4 polynucleotide kinase (Invitrogen) and self-ligated by T4 DNA ligase (Invitrogen). All constructs were verified by DNA sequencing.

pMlK-Neo-mYes was a gift from Marius Sudol (Addgene plasmid #18067). SYF cells were transfected 24 h before performing experiments using Lipofectamine 3000 (Invitrogen) according to manufacturer’s instructions.

### Integrin Activation Assay

Cells were serum starved for 2 h, trypsinised, and resuspended in serum-free medium containing 0.25 mg/ml soybean trypsin inhibitor (Sigma) and 0.1 mg/ml BSA, as previously described^10^. After centrifugation, cells were suspended in serum-free medium with 0.1 mg/ml BSA at 37 °C for 30 min to recover.

Cells were incubated for 30 min at 37 °C with 1–3 mM MnCl_2_ (Mn^2+^). For inhibitor assays, cells were first incubated for 30 min at 37 °C with 1–100 μM pervanadate (PV)^54^, or left untreated. Subsequently, cells were incubated for 30 min at 37 °C in the absence or presence of 3 mM MnCl_2_. Cells were lysed, and analysed by Western blot (WB).

Adhesion to A12-Dis^55^ or to the third fibronectin type III repeat of tenascin-C with 848RGD mutated to the α9β1 specific RAA (TNfn3RAA, as described^10^) was performed in the absence or presence of 1 mM MnCl_2_ for 90 min. Cells were then washed with ice-cold TBS and lysed with RIPA buffer (50 mM Tris pH 7.6, 150 mM NaCl, 1% Triton-X-100, 0.5% sodium deoxycholate (DOC), 0.1% SDS, 1 mM EDTA, 1x complete protease inhibitor cocktail (Roche Applied Science), 25 mM NaF, 1 mM Na_3_VO_4_, and 20 mM β-glycerophosphate). Laemmli sample buffer was added to the samples and then heated to 100 °C. Samples were analysed by WB.

### Immunoprecipitation of  Tyrosine Phosphorylated Proteins

Cell suspensions prepared as described for the integrin activation assay were washed in ice-cold TBS containing 25 mM NaF, 1 mM Na_3_VO_4_, and 20 mM β-glycerophosphate, lysed in TBS containing 25 mM NaF, 1 mM Na_3_VO_4_, 20 mM β-glycerophosphate, and 1% Triton X-100, 0.5 mM EDTA, 1x Halt complete protease inhibitor cocktail (Roche Applied Science), and 1x Phosphatase Inhibitor Cocktail (Thermo Scientific). Cell extracts were clarified by centrifugation for 2.5 min at 13,200 rpm. SDS was added to cell extract for a final concentration of 0.25% and boiled for 5 min, as appropriate, since PY99 efficiently recognised phosphorylated tyrosine on denatured proteins as suggested by preliminary data. Lysates were incubated with 20 μl Protein G Sepharose 4 Fast Flow beads (GE Healthcare) coated with PY99 (10 μg antibody/20 μl beads) for 60 min at 4 °C. Beads with cell extract were washed three times with lysis buffer containing 0.25% SDS, as appropriate, and twice in lysis buffer without SDS. Tyrosine phosphorylated proteins on beads were eluted from beads by the addition of 0.2 M phenyl phosphate, a phosphotyrosine mimic^56^,^57^ for 2 h at 4 °C. Samples were collected by centrifugation, boiled after adding sample buffer, and analysed by WB.

### Western Blotting

WB was performed as described previously^10^ and quantified using NIH image, version 1.61, or, in some cases, signals were acquired by western blot imaging system C-Digit (LI-COR Bioscience, USA) and analysed by Image Studio Digits Ver 4.0 (LI-COR).

### Fluorescence Activated Cell Sorting (FACS)

FACS was performed as described previously^10^, with minor modifications. For cell surface integrin expression, untreated cells and cells treated with appropriate IgG control or secondary antibody only served as negative controls. Suspended G361 cells were fixed in 1% paraformaldehyde (PFA) for 7 min. For validation of α9 and α4 integrin knockdown, cells were stained with primary and secondary antibodies, before fixation with 1% PFA for 7 min followed by wash and resuspension in FACS buffer for analysis. The mean of the fluorescence intensity detected by 12G10 (active β1) staining was normalised to MAB1959 (total β1 levels) staining.

### Fibronectin Matrix Assembly

FN matrix assembly assays were performed as described previously^16^, with minor modifications. Glass coverslips (12 mm) were coated with 10 μg/ml A12-Dis or TNfn3RAA. 3.8 × 10^5^ cells were seeded in complete medium overnight at 37 °C. Fresh complete medium alone or containing 0.5 mM MnCl_2_, 20 μg/ml TS2/16, 0.5 mM MnCl_2_ and 10 μg/ml Y9A2 was added and the cells were incubated at 37 °C for 8 h. Immunofluorescence microscopy was performed as described previously^10^,^58^. Micrographs were taken of five random areas of the coverslip for quantification. Controls for nonspecific cross-reaction of secondary antibody were included and gave no staining above background. Micrographs were acquired on a Zeiss Axioplan-2 microscope (Carl Zeiss, Inc.) equipped with a Cool SNAP camera, using Zeiss Plan-NEOFLUAR 10x NA 0.30 and 20x NA0.05 objectives, or on a BZ-9000 (Keyence, Japan, Objectives: Plan Apo 20x/0.75, Nikon, Japan). A threshold was set to measure fluorescence intensities above background levels and then the images were processed using MetaMorph software (Molecular Devices), Adobe Photoshop (Adobe), and ImageJ (NIH).

### Deoxycholate (DOC) Insolubility Assay

DOC fractionation of FN was carried out as described previously (Sechler *et al*.) with a minor modification. Briefly, G361 cells (8 × 10^5^ cells) in complete medium were seeded on RAA-coated 12 well plates and left overnight at 37 °C. Cells were lysed with DOC lysis buffer, and precipitates after centrifugation was collected as DOC insoluble fractions. cFN in DOC insoluble fractions was analysed by WB with anti-EDA FN (FN-3E2). β-actin in total cell lysates were analysed as loading controls. Signals were acquired by C-Digit and analysed by Image Studio Digits Ver 4.0 as described above.

### Immunocytochemistry

Immunocytochemistry was performed as described previously^10^ with minor modifications. Cells were seeded on glass coverslips coated with 10 μg/ml A12-Dis or with 10 μg/ml TNfn3RAA for 60 min and for another 30 min in the absence or presence of 1 mM MnCl_2_ at 37 °C. F-actin was visualised by phalloidin 647, Invitrogen. Micrographs were acquired on an Axiovert 200 M LSM 520 (Carl Zeiss) using a Zeiss C-Apochromat x63 NA 1.4 oil objective. Images were processed using ZEN 2009 (Carl Zeiss) and ImageJ.

### Cell Migration Assays

Cell migration assays were performed as described previously^10^, with minor modifications. 0.75–1 × 10^5^ cells in 100 μl of serum free medium containing 0.1 mg/ml BSA were seeded on inserts. After fixation by 2% glutaraldehyde, filters were washed with PBS and stained using 1% crystal violet. Filters were washed with PBS before eluting with 1% SDS/PBS for 30 min. The optical densities (o.d.) of the eluted fractions were measured at 590 nm.

### Statistical Analysis

Unless otherwise indicated, data are presented as mean ± s.e.m. from three or more independent experiments; the statistical significance of differences between groups was calculated using GraphPad Prism 6.0 (GraphPad Software Inc.) or SigmaPlot (Systat Software Inc.). Differences were considered to be significant when *P* < 0.05 (*), *P* < 0.01 (**), or *P* < 0.001 (***). WBs were performed at least three times and representative examples are presented.

## Additional Information

**How to cite this article**: Høye, A. M. *et al*. The Phosphorylation and Distribution of Cortactin Downstream of Integrin α9β1 Affects Cancer Cell Behaviour. *Sci. Rep.* **6**, 28529; doi: 10.1038/srep28529 (2016).

## Supplementary Material

Supplementary Information
