# Supplementary material for: The Phosphorylation and Distribution of Cortactin Downstream of Integrin α9β1 Affects Cancer Cell Behaviour
Source: Sci Rep. 2016 Jun 24;6:28529. doi: 10.1038/srep28529 (PMC4919783; doi:10.1038/srep28529)

Supplementary Information

The Phosphorylation and Distribution of Cortactin Downstream of Integrin $\alpha9\beta1$ Affects Cancer Cell Behaviour.

Anette M. Høye[1], John R. Couchman[1], Ulla M. Wewer[1], and Atsuko Yoneda[1,2*].

[1]Department of Biomedical Sciences, Faculty of Health and Medical Sciences, and Biotech Research and Innovation Centre (BRIC), University of Copenhagen, Copenhagen Biocenter, Ole Maaløes Vej 5, 2200 Copenhagen N, Denmark.

[2]Laboratory of Genome and Biosignals, Tokyo University of Pharmacy and Life Sciences, 1432-1 Horinouchi, Hachioji-city, Tokyo 192-0392, Japan.

*Corresponding author (present address): Atsuko Yoneda, Laboratory of Genome and Biosignals, Tokyo University of Pharmacy and Life Sciences, 1432-1 Horinouchi, Hachioji-city, Tokyo 192-0392, Japan. Tel: +81 42 676 7221; Fax: +81 42 676 7249, E-mail: ayoneda@toyaku.ac.jp

Supplementary Figure 1. G361 cells utilise cFN to assemble FN matrix.

A) RT-PCR for specific detection of EDA+ FN in G361 cells was performed as described[59]. * indicates EDA+ FN (374 bp) and ** indicates EDA- FN (104 bp). GAPDH and single FN primers (fwd and rev respectively) were included as controls. **B)** A gelatin agarose column was used to deplete pFN from serum. Representative micrographs of G361 cells attached to A12-Dis in the absence or presence of Mn^{2+} (0.5 mM) and stained for FN. Scale bars = 100 µm. **C)** WB of untreated or Mn^{2+} treated G361 cell showing the DOC insoluble fraction analysed with a FN EDA specific antibody. TCL analysed for actin was included as loading control. **D)** Quantitation of **C)**. Mn^{2+} treatment significantly increases the amount of DOC-insoluble cFN.

Supplementary Figure 2. **Immunoprecipitation of candidate proteins.**

IP of tyrosine phosphorylated proteins using anti-pTyr antibody PY99 of G361 TCLs stimulated with Mn^{2+} were analysed by WB for candidate proteins: SLP76, Raf1, numb, and paxillin as indicated. TCL, preclear beads, and antibody (Ab) alone were included as controls.

Supplementary Figure 3. FAK depletion does not decrease cortactin phosphorylation in G361 cells.

A) Kinases present in G361 cells with the potential to phosphorylate cortactin tyrosine residues. Untreated or Mn^{2+} stimulated G361 TCLs were analysed for the presence of FAK (125 kDa), PYK2 (120 kDa), Arg/ Abl2 (128 kDa), Yes (62 kDa), Crk (42 kDa), v-Src, Fyn (59 kDa), and Hck (60 kDa) kinases by WB as indicated. **B)** FAK depletion does not decrease pp75 or cortactin Y470 phosphorylation in G361 cells.

TCL of G361 cells transfected with NC or FAK siRNA in suspension treated with Mn^{2+} (3 mM). Samples were analysed by WB and probed for antibodies as indicated.

Supplementary Figure 4. Cortactin KO WT and MEFs can assemble FN matrix. **A)** Micrographs of WT or cortactin KO MEFs attached to A12-Dis, in the absence or presence of Mn^{2+} (0.5 mM) and stained for FN. **B)** Quantitation of **A)**. Mean of data from WT MEFs was set at 1.0.

Supplementary Figure 5. Expression of Y421 and Y470 Cortactin mutated proteins in G361 migration assays.

Parental G361 were either treated with NC or CTTN siRNA. Migration of G361 cells stably transfected with WT-myc, Y421F-myc, Y470F-myc, Y421D-myc, or Y470D-myc where indicated. All stable cell lines were double transfected with CTTN siRNA and their respective plasmid 48 hours before migration. TCLs were analysed by WB and probed for cortactin (ab11066), myc, and actin.

Supplementary Figure 6. Phosphorylation of cortactin Y470 is modified by α9β1 and regulation of phosphatase activity.

A-B) Representative FACS profiles of α9β1 and α4 in G361. G361 cells were treated with the siRNAs indicated and stained for cell surface levels of α9β1 (Y9A2 antibody) in **A)** or α4 (HP1/2 antibody) in **B)**. **C)** Effect of α4 and α9 knockdown on tyrosine phosphorylation. G361 cells were either treated with NC, α9, or α4 siRNA, and cells in suspension were then untreated or treated with Mn^{2+}. TCL was analysed by WB. Short and long exposure times of WBs are shown for pY470. **D)** G361 cells in suspension were untreated or treated with Mn^{2+} (3 mM) in the absence or in the

presence of 1 μM or 10 μM pervanadate (PV). TCLs were analysed by WB for pTyr (4G10), pY470 cortactin, pY421 cortactin, cortactin, pY416 SFK, Yes kinase, and actin, as indicated.

Supplementary Figure 7. α9β1 related signalling events in high α9β1 expressing cells.

We hypothesise that α9β1 **(1)**, in high α9β1 expressing cells, exerts inhibition of PTEN when in a low/intermediate active state. **(2)** PTEN has been shown to regulate Rac1-GTP levels[58] and we have previously shown that α9β1 integrin adhesion and migration on A12-Dis and TNfn3RAA are dependent on Rac1-GTP[10]. Whether this is dependent on PTEN is not currently known. Here we show that PTEN affects phosphorylation of cortactin Y470, and thereby its cellular distribution. Upon activation of α9β1, a SFK is phosphorylated on Y416 **(3)**. Phosphorylation of Y416 activates SFK kinase activity. Next, the activated SFK phosphorylates cortactin at Y470, which is recruited to FAs where it can regulate cell migration **(4)**. Our data also suggest that cortactin may have an inhibitory effect on the activation state of β1 integrins **(5)** in G361 cells. Finally, integrin activation by α9β1-ligands leads to increased FN matrix assembly **(6)**.

Supplementary Figure S1

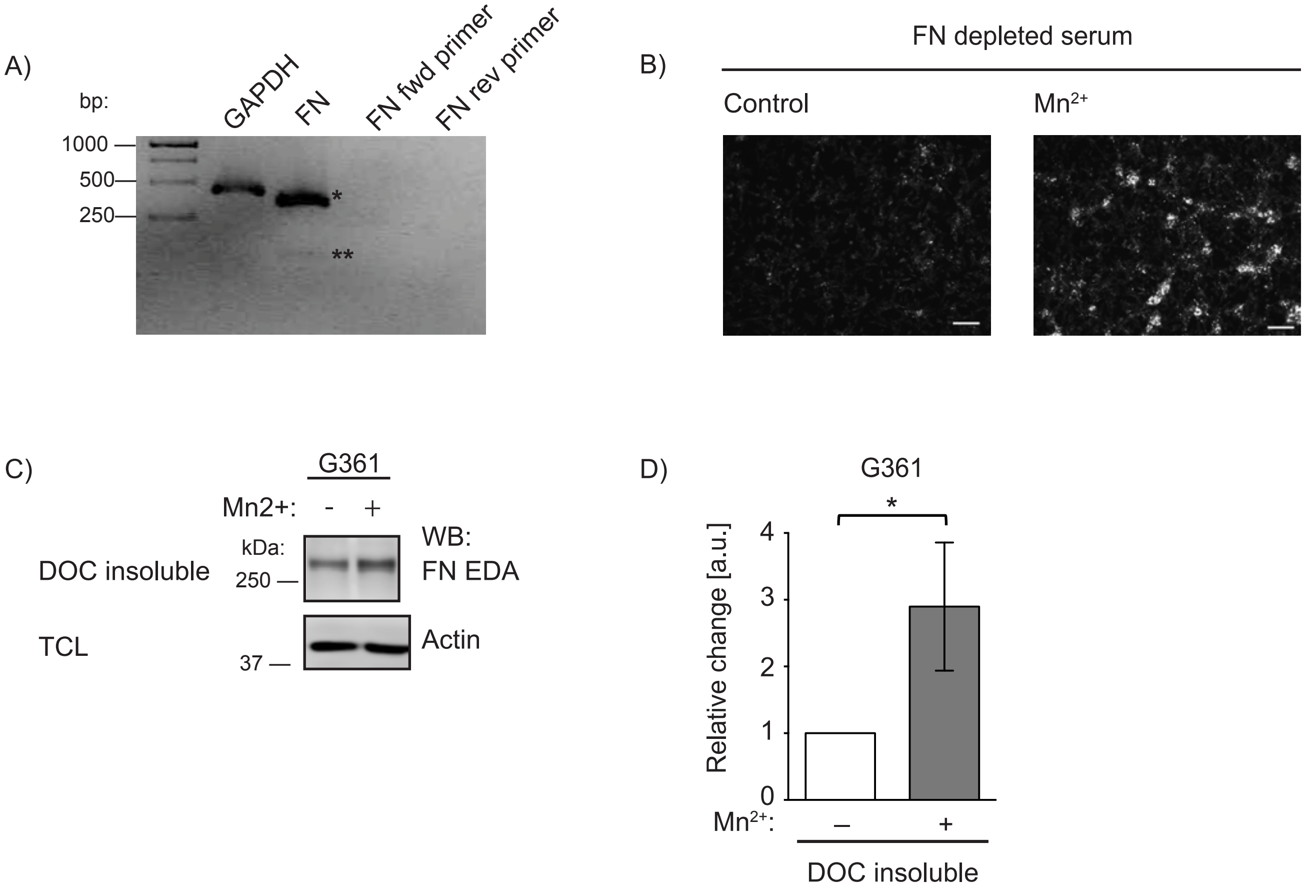

Supplementary Figure S2

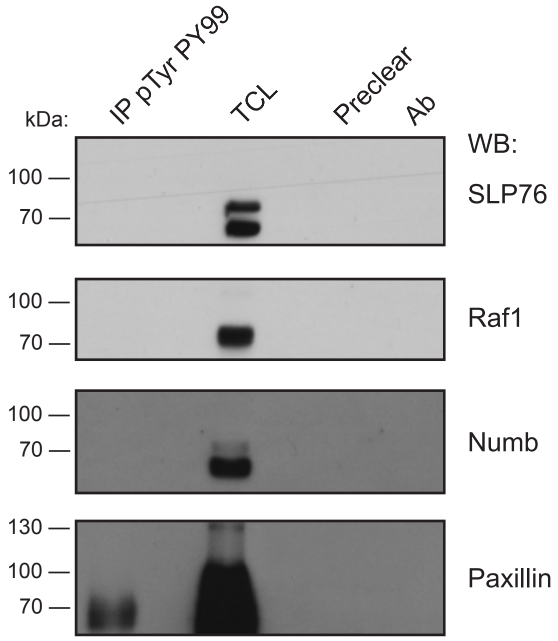

Supplementary Figure S3

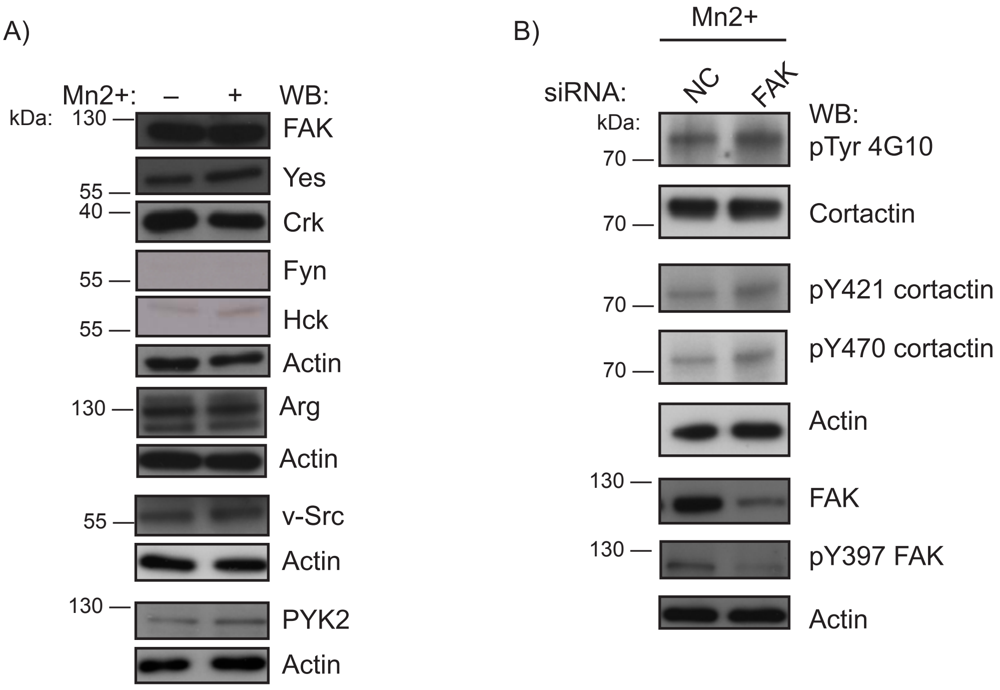

Supplementary Figure S4

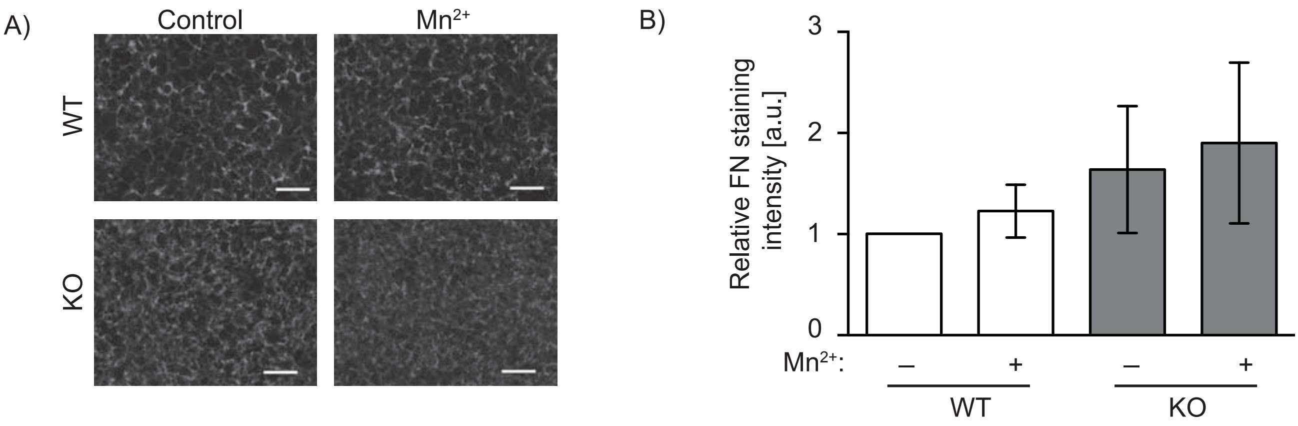

Supplementary Figure S5

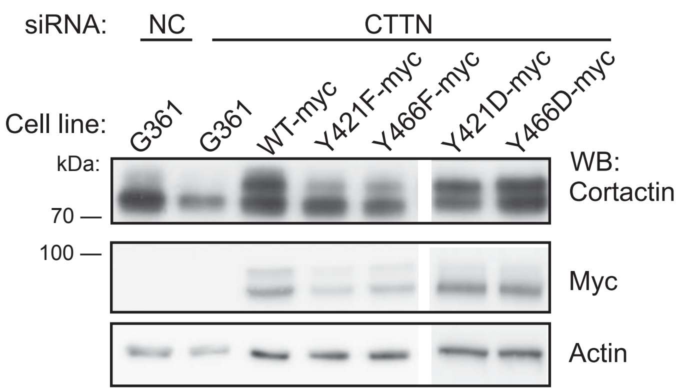

Supplementary Figure S6

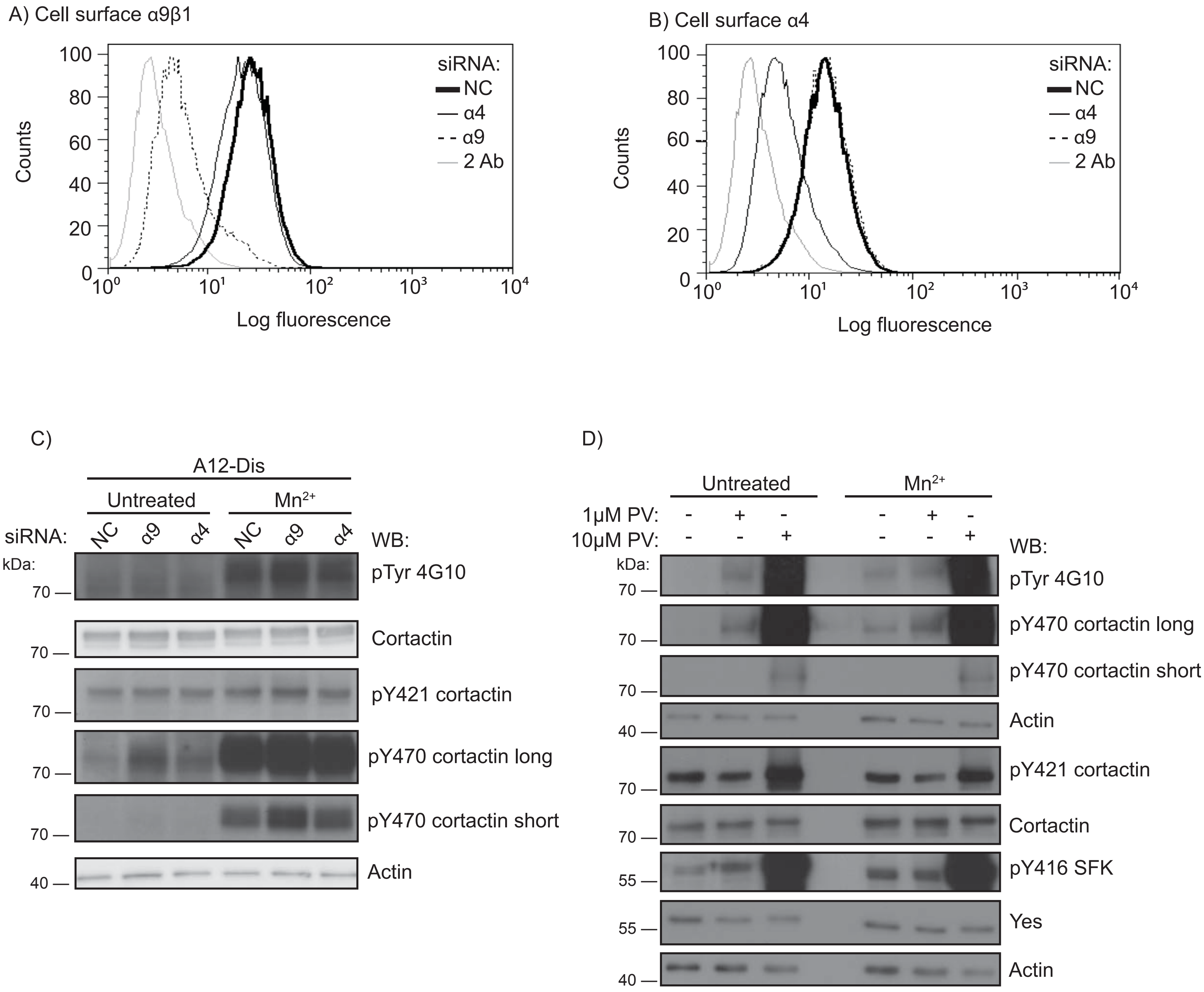

Supplementary Figure S7

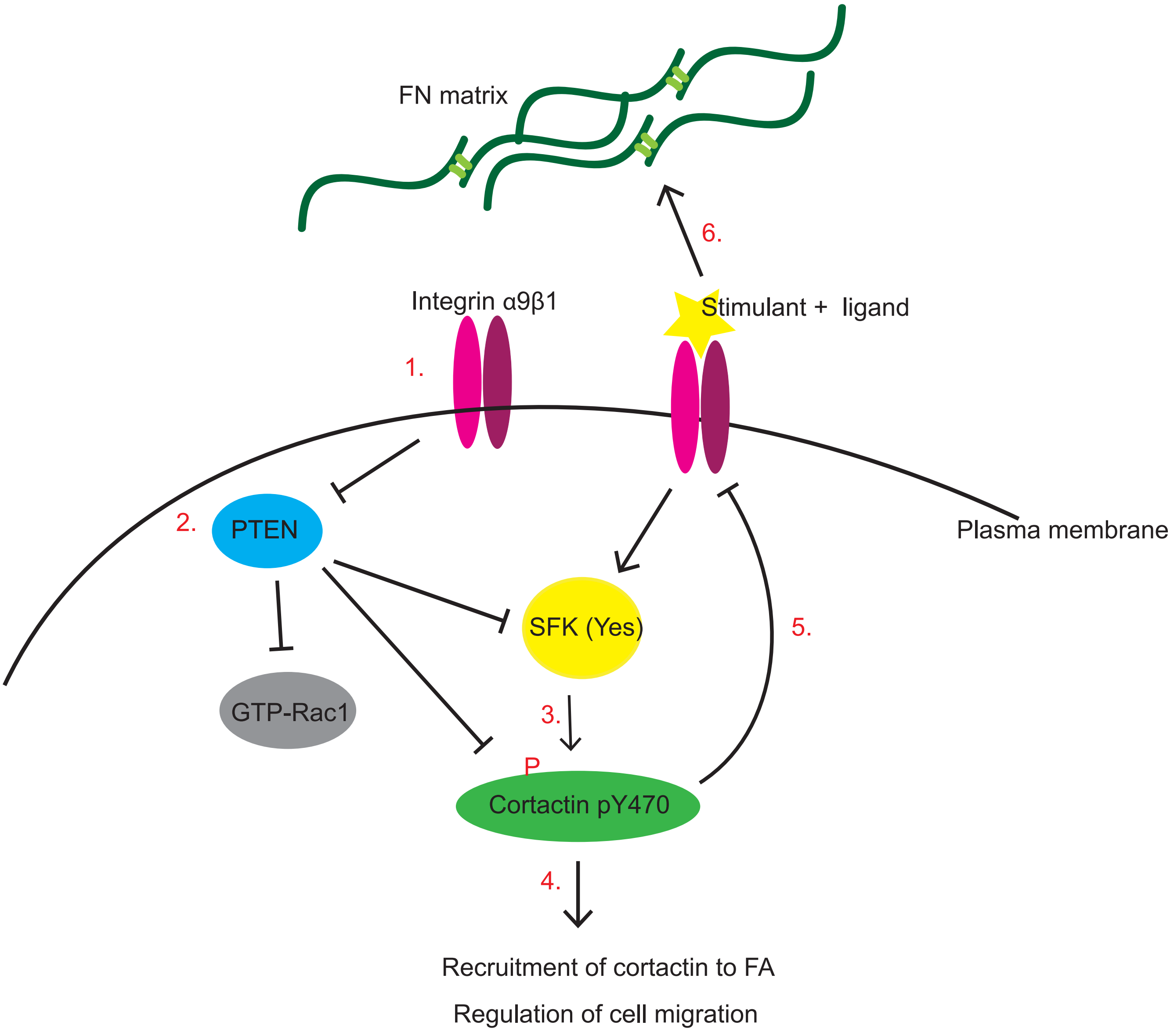

Supplement: Supplementary Information [file srep28529-s1.pdf]
